# Immune checkpoint inhibitor-induced diarrhea and colitis: an overview

**DOI:** 10.1007/s00520-024-08889-2

**Published:** 2024-09-23

**Authors:** Marianne Zoghbi, Kathryn J. Burk, Elio Haroun, Maria Saade, Maria Teresa Cruz Carreras

**Affiliations:** 1 Department of Leukemia, The University of Texas MD Anderson Cancer Center, Houston, TX USA; 2 Department of Emergency Medicine, The University of Texas MD Anderson Cancer Center, Houston, TX USA; 3 Faculty of Medicine, Saint Joseph University of Beirut, Beirut, 1100 Lebanon

**Keywords:** Immune checkpoint inhibitors, Anti-CTLA4, Anti-PD1, Immune-related adverse events, Colitis, Diarrhea

## Abstract

Immune checkpoint inhibitors (ICIs) have emerged as an integral component of the management of various cancers and have contributed to significant improvements in overall survival. Most available ICIs target anti-cytotoxic T-lymphocyte-associated protein 4 (anti-CTLA4), and anti-programmed cell death 1/programmed cell death ligand 1 (anti-PD1/PDL1). Gastrointestinal immune-related adverse events remain a common complication of ICIs. The predominant manifestations include diarrhea and colitis, which often manifest concurrently as immune-mediated diarrhea and colitis (IMDC). Risk factors for developing these side effects include baseline gut microbiota, preexisting autoimmune disorders, such as inflammatory bowel disease, and type of neoplasm. The hallmark symptom of colitis is diarrhea which may be accompanied by mucus or blood in stools. Patients may also experience abdominal pain, fever, vomiting, and nausea. If not treated rapidly, ICI-induced colitis can lead to serious life-threatening complications. Current management is based on corticosteroids as first-line, and immunosuppressants like infliximab or vedolizumab for refractory cases. Microbiota transplantation and specific cytokines and lymphocyte replication inhibitors are being investigated. Optimal patient care requires maintaining a balance between treatment toxicity and efficacy, hence the aim of this review is to enhance readers’ comprehension of the gastrointestinal adverse events associated with ICIs, particularly IMDC. In addition to identifying the risk factors, we discuss the incidence, clinical presentation, workup, and management options of IMDC.

## Introduction

Immune checkpoint inhibitors (ICIs) have emerged as an integral component of the management of various cancers and have contributed to significant improvements in overall survival (OS) and progression-free survival (PFS) across diverse clinical settings [1]. Most commercially available ICIs target anti-cytotoxic T-lymphocyte-associated protein 4 (anti-CTLA4), e.g., ipilimumab; anti-programmed cell death protein 1 (anti-PD1), e.g., pembrolizumab, and nivolumab; or anti-programmed death-ligand 1 (anti-PDL1), e.g., atezolizumab [2]. By targeting regulatory immune checkpoint molecules, these monoclonal antibodies boost T cell-mediated antitumor immunity and enhance the immune-mediated clearance of tumor cells [3].

Although ICIs can effectively target tumor cells, they may also disrupt immune homeostasis in non-tumor tissues, leading to a spectrum of immune-related adverse events (irAEs), which primarily affect the skin, gastrointestinal tract, lungs, and endocrine glands [4]. Among these, gastrointestinal irAEs are the second most prevalent adverse effects of ICI therapy, accounting for 35–50% of all ICI-induced adverse events [2]. The predominant manifestations of gastrointestinal irAEs include diarrhea and colitis, which often manifest concurrently as immune-mediated diarrhea and colitis (IMDC) [5].

Optimal patient care requires maintaining a balance between treatment toxicity and efficacy. The aim of this review is to enhance readers’ comprehension of the gastrointestinal irAEs associated with ICIs, particularly IMDC. In addition to identifying the risk factors for these adverse events, we discuss the incidence, clinical presentation, and management of IMDC. Because gastrointestinal irAEs can manifest anywhere along the gastrointestinal tract, we also provide a brief overview of other potential gastrointestinal manifestations of ICIs.

## Epidemiology

Around 85% of patients on CTLA4 inhibitors and 75% on PD1 inhibitors report irAEs, which have a longer duration and mild-to-moderate severity compared with chemotherapy-related adverse events and can occur even after therapy conclusion [6]. Gastrointestinal irAEs, such as esophagitis, gastritis, aphthous ulcers, and diarrhea, are prevalent, with ICI-induced colitis being among the most common irAEs [5]. The incidence and severity of diarrhea are higher among patients receiving anti-CTLA4 agents than among those receiving anti-PD1 agents [2, 5]. Combination therapy intensifies diarrhea and colitis frequency and severity, potentially causing rarer toxicities. The incidence of diarrhea is 12.1–13.7% for patients receiving PD1 inhibitors, and 30.2–35.4% for patients receiving CTLA4 inhibitors. [7]. Notably, the incidence of grade 3 or 4 diarrhea among patients receiving anti-CTLA4 therapy (10%) is higher than that among patients receiving anti-PD1/PDL1 agents (1–2%) [8]. The incidence of any grade IMDC with ipilimumab and nivolumab combination is close to 40–50%.

Patients receiving ipilimumab manifested a higher risk of toxicity with dose elevation. This is demonstrated by comparing adverse events in patients receiving 3 mg/kg versus 10 mg/kg of ipilimumab, where the risk of severe-grade enterocolitis was 7% versus 25%, the incidence of diarrhea was 6% versus 10%, and the incidence of colitis was 2% versus 5% [2, 9]. No dose-dependent toxicity is observed with nivolumab or pembrolizumab [10]. One possible explanation is that all currently used doses saturate the receptors completely.

In a 2019 systematic review, Arnaud-Coffin et al. reported that 11% of patients on PDL1 inhibitors and 36% of patients on CTLA4 inhibitors developed diarrhea, which was the most common adverse event of any grade [11]. A review study from MD Anderson Cancer Center similarly reported that 36% of patients had ICI-induced diarrhea, with 24% requiring immunosuppressive treatment [12]. In a 2017 review of 34 studies, the incidence of any-grade colitis was 2.4%, that of grade 3 or 4 colitis was 1.7%, and that of grade 3 or 4 diarrhea was 2.3% [13].

## Pathogenesis overview

The mechanism of ICI-induced colitis seems to be linked to autoimmune events. Although the pathogenesis is not yet fully understood, key factors include T cell hyperactivation, increased memory T cells, lymphocyte infiltration, cytokine activation, and changes in gut microbiota-immune system interactions. ICI-induced colitis often involves significant infiltration of CD4 + and CD8 + T cells, with CD8 + T cells likely originating from tissue-resident memory T cells (TRM), leading to early symptom onset after treatment initiation [7]. Studies highlight that activated CD8 + TRM cells at the lamina propria-epithelial interface drive cytotoxicity in ICI-induced colitis and ulcerative colitis (UC) [14]. Evidence suggests that CD8 + TRM cells are key drivers of inflammation, possibly playing a larger role in ICI colitis than inflammatory bowel disease (IBD). Notably, CD8 + TRM cells are more abundant and activated in ICI-induced colitis than in UC. Patients taking ICI who develop colitis show reduced TRM cells compared to other patients under ICI without colitis [7].

However, studies have shown a lack of correlation between CD8 + T cell cytotoxicity and clinical and histological colitis severity, suggesting that CD8 + T cell granzyme B levels might indicate only the initiation of inflammation. Other factors (outside CD8 + T cell biology) likely contribute to determining clinical severity and the necessity for increased immunosuppressive therapy [15].

Furthermore, CTLA-4 is overexpressed in regulatory T (Treg) cells that help maintain immune tolerance. Anti-CTLA-4 drugs inhibit CTLA-4 + Treg cells in tumors and intestines, leading to the activation of effector T cells like cytotoxic T-lymphocytes (CTLs) and resulting in ICI colitis [16].

## Risk factors

### Gut microbiome

Recent evidence indicates that the composition of a patient’s intestinal microbiota significantly influences both the effectiveness of ICI immunotherapy and the risk of adverse events, though this role is not yet fully understood. Several studies have explored the link between microbial signatures and response to ICI treatment, revealing notable differences in microbial composition between responders and non-responders [17]. However, results often conflict regarding which specific commensals correlate with clinical response to ICIs. These inconsistencies may stem from patient-specific microbiota, varying study designs, and results analysis. A recent systematic review found that the presence of B. *longum* and F. *prausnitzii* is associated with better ICI responses [18]. Larger, multi-center studies are needed to validate these findings. In this context, ongoing studies (e.g., NCT03643289, NCT04107168, and NCT05037825) aim to clarify how microbiota and their byproducts impact cancer therapies.

### Autoimmune conditions

ICI therapy can exacerbate preexisting autoimmune conditions. However, in such patients, immune-mediated toxicities are usually mild and can be managed without therapy discontinuation. Dual therapy with different types of ICIs also increases the risk of irAEs [8].

Several case series have shown that individuals with preexisting autoimmune disorders, such as IBD, have an increased risk of ICI-induced colitis. Patients with IBD appear to tolerate anti-PD1 therapy better than anti-CTLA4 therapy [19].

### Type of cancer treatment

The nature of the neoplasm and the type of treatment administered may also influence the risk of ICI-induced colitis, particularly in melanoma patients. Notably, patients with stage 3 neoplasms have a higher incidence of colitis than those with stage 4 neoplasms [20]. The most plausible explanation for this difference is that stage 3 melanoma is treated with a higher ipilimumab dose than stage 4 (metastatic) melanoma: 10 mg/kg for stage 3 versus 3 mg/kg for metastatic. Furthermore, melanoma patients are generally more exposed to ipilimumab treatment regimens and higher doses than patients with other cancers. This may explain why compared to patients with non-small cell lung cancer or renal cell carcinoma, melanoma patients have almost twice the incidence of any grade colitis and grade 3 or 4 colitis [13].

### Genetics and biomarkers

Increased serum levels of IL-17 and eosinophils may increase the risk of gastrointestinal irAEs [21]. Also, human leukocyte antigen–DQB1*03:01 has been associated with the onset of ICI-induced colitis [12]. However, a specific gene or biomarker predicting the development of IMDC has not been discovered yet.

## Clinical presentation

The primary symptom of colitis is diarrhea, which is often accompanied by blood, but the absence of diarrhea does not rule out colitis. Other symptoms include mucus in stools, abdominal pain, fever, vomiting, and nausea [5]. Variants of colitis in the upper gastrointestinal tract include gastritis, gastric ulcerations, duodenitis, and esophagitis [22].

ICI-induced colitis can progress rapidly, leading to serious complications such as toxic megacolon, ileus, peritonitis, bowel perforation, and death [23]. Historically, the classification of IMDC is based on a grading system from 1 to 5, established for oncology clinical trials called the Common Terminology Criteria for Adverse Events (CTCAE), although the clinical utility of this grading system has not been established. Briefly, grade 1 colitis is defined as asymptomatic, with pathologic or radiographic findings only; grade 2, as abdominal pain, with mucus or blood in the stool; grade 3, as additional fever or peritoneal signs; grade 4, as perforation, ischemia, necrosis, or toxic megacolon; and grade 5, as death. Grade 1 diarrhea is defined as an increase of less than four stools per day over baseline; grade 2, as an increase of 4–6 stools; grade 3, as an increase of 7 or more stools per day, with hospitalization indicated; grade 4, as life-threatening with urgent intervention indicated; and grade 5, as death [24].

IMDC typically manifests 5–10 weeks after the second or third dose of treatment, but studies suggest that IMDC can develop even 4 months after the last dose and potentially recur 1–2 years after treatment discontinuation [5].

## Diagnosis

### Stool and serologic markers

Patients presenting with diarrhea should undergo a comprehensive set of laboratory tests to investigate the possibility of concurrent conditions, such as celiac disease, anemia, electrolyte abnormalities, low albumin levels, or treatment-related thyroid toxicities. Hence, the work-up should include tissue transglutaminase immunoglobulin A, C-reactive protein, complete blood count, metabolic panel, albumin, and thyroid-stimulating hormone tests [25].

Disease progression may manifest as increased levels of inflammatory markers, such as C-reactive protein. In addition, stool should be examined for infectious pathogens like *Clostridium difficile*, *Cytomegalovirus*, and *Salmonella,* as ICI-induced colitis and infection can co-exist [26]. A pancreatic elastase test can be done to evaluate exocrine pancreatic insufficiency. Fecal calprotectin, a serologic marker with high sensitivity and specificity for intestinal inflammation, can help predict disease activity, but it is not specific to ICI-induced colitis [8]. Rather, it serves as a noninvasive biomarker of endoscopic and histologic remission, potentially reducing the need for invasive procedures. Moreover, fecal lactoferrin testing can guide endoscopy prioritization, as fecal lactoferrin is strongly correlated with the inflammation observed on endoscopy, and can guide the histological evaluation of biopsy specimens [27].

### Endoscopic features

There is no correlation between reported clinical symptoms and endoscopic findings in patients with ICI-induced colitis [28]. However, having an endoscopy within a week of symptoms was correlated with a shorter duration of steroid therapy and a shorter duration of symptoms. The gold standard for diagnosing and staging colitis is ileocolonoscopy with biopsy; however, because the left colon is affected in 98% of cases, flexible sigmoidoscopy may be sufficient for diagnosis. High-risk endoscopic features, such as deep ulcers and extensive colonic involvement, are observed in 39% of patients. Identifying these features is crucial, as they are associated with an increased demand for biologic therapy and prolonged hospitalization [29]. In 36–42% of cases, non-ulcerative features like erythema, erosions, edema, and exudate are observed. Pancolitis, a predictor of steroid-refractory colitis, is reported in 23–40% of cases. Left-sided colitis and ileitis are observed in 31–43% and 11–14% of cases, respectively [29]. A normal-appearing mucosa on endoscopic examination does not exclude enterocolitis [8]; indeed, 37% of patients have a normal colonoscopy, and 15% have normal histology [29]. Normal histology is most likely reflective of enteritis rather than colitis leading to the symptoms, as demonstrated in several studies.

### Histology

The common histologic features of ICI-induced colitis include acute colitis indicators, such as increased lamina propria cellularity, intraepithelial neutrophilic infiltrates, cryptitis, crypt abscesses, and apoptotic cells, which may precede diarrhea onset [8]. Chronic colitis features, such as intraepithelial lymphocytes, basal lymphocytes, and crypt distortion, may also be present [8]. The pattern of ICI-induced colitis can vary by drug, with ipilimumab linked to diffuse active colitis and nivolumab associated with lymphocytic colitis and, with long-term treatment, chronic active colitis [30]. No single criterion confirms the diagnosis, emphasizing the need for contextual interpretation in clinical assessments.

### Imaging

Limited data suggests that imaging, particularly computed tomography (CT), may serve as an alternative for diagnosing ICI-induced colitis. Recent studies have shown that CT has suboptimal sensitivity (74%) and specificity (50%) when correlating radiologic findings with correct histological diagnosis of enterocolitis [31]. Despite these limitations, CT scans remain helpful for excluding complications and assessing disease severity, especially if toxic megacolon, perforation, abscess, or obstruction is suspected [32]. Further exploration of pathognomonic radiological signs is needed to consider CT as part of the standard of care for diagnosing ICI-induced colitis as it offers the advantage of being less invasive than endoscopy with biopsy.

### Differential diagnosis

In patients receiving ICI therapy, the initial step of the differential diagnosis involves ruling out infectious causes (viral, bacterial, or parasitic) of diarrhea and abdominal pain. Microbiological studies and stool cultures are crucial in this context, considering these patients’ increased risk of infection [5, 8]. Distinguishing between infectious and ICI-induced colitis can be challenging, but specific features caused by pathogens can aid in differentiation. Opportunistic infections like *Cytomegalovirus*, diagnosed by characteristic cellular and intranuclear inclusions, can complicate ICI-induced colitis and impact steroid response [33]. *C. difficile* infections overlaid on ICI-induced colitis have been documented in patients receiving ICI therapy [34].

Furthermore, gastrointestinal metastases as a potential cause of colitis must be excluded, particularly in patients with clinical signs of peritonitis, which warrants evaluation with CT to rule out colonic perforation [8].

Nonsteroidal anti-inflammatory drugs can induce conditions like lymphocytic colitis and collagenous colitis, which can mimic ICI-induced colitis. The harm these conditions impose on the colon includes erosions and ulcers, with distinct histological findings [35].

Distinguishing between ICI-induced colitis and IBD is crucial, and distinctive histological features favor either diagnosis. ICI-induced colitis often presents with a rapid onset of symptoms, whereas IBD has an insidious onset, and specific lesions like fistulas may suggest *C. difficile* in patients with IBD [36].

## Management

If not treated rapidly, ICI-induced colitis can lead to serious life-threatening complications. Treatment primarily includes corticosteroids and biologic medications like infliximab or vedolizumab. Other treatments being investigated include fecal microbiota transplantation and the use of inhibitors of specific cytokines and lymphocyte replication. Table 1 provides a summary of the available treatment options.

**Table 1:** Treatment options for immune checkpoint inhibitors-induced colitis

Category	Agents	Use
**Corticosteroids**	Budesonide	First-line
	Prednisone	
**Immunosuppressants**	Infliximab	If recurrence after corticosteroids
	Vedolizumab	
	Mycophenolate mofetil	In addition to corticosteroids Reduce treatment duration—investigational
**Microbiota transplantation**		Refractory to corticosteroids and immunosuppressants—investigational
**Alternative options**	Janus kinase inhibitors (Tofacitinib)	Investigational
	Calcineurin inhibitors (Tacrolimus)	
	Anti–IL-12/IL-23 (Ustekinumab)	
	Anti IL-1 (Anakinra)	

### Corticosteroids

First-line therapy relies on corticosteroids. However, these agents have undesirable side effects, especially when they are used for extended periods. Therefore, it is crucial to taper them over 4–6 weeks as soon as symptom improvement is noted [5] to avoid serious adverse events such as infections, glucose intolerance, and impaired bone metabolism [37]. Following first-line therapy with corticosteroids, CTLA4 inhibitor–induced colitis recurs in about 44% of patients, and PD1/PDL1 inhibitor–induced colitis recurs in about 34% of patients [7]. In patients with recurrent colitis, the introduction of supplementary immunosuppressants, such as infliximab and vedolizumab, is recommended [5].

### Immunosuppressants

Infliximab is more commonly used as the primary immunosuppressive agent [38], whereas vedolizumab, which is less studied, is recommended for patients with contraindications or nonresponse to infliximab [39].

Infliximab, a monoclonal antibody-targeting tumor necrosis factor α (TNF-α), has elicited remission rates of up to 71% in cases of corticosteroid-refractory colitis. Infliximab is administered intravenously at a dose of 5 mg/kg and typically induces a rapid response within days [40]. A single administration is sufficient in about 70% of patients, but some require additional doses [41]. In that case, patients are offered a 3-dose regimen at weeks 0, 2, and 6. Compared with steroids, the early administration of infliximab leads to a shorter time to symptom resolution [42]. However, studies of infliximab as a first-line therapy for ICI-induced colitis are lacking.

Abundant preclinical data indicate that TNF blockade synergizes with anti-PD-1 to enhance immune responses in solid tumors, thereby improving antitumor immunity. A study showed that TNF expression is positively associated with expression of PD-L1 in human melanoma specimens indicating that TNF blockade could potentially prevent PD-L1 expression. This finding supports the development of combination therapy involving anti-PD-1 and anti-TNF for cancer patients [43].

Vedolizumab, an alternative to infliximab, is a humanized monoclonal immunoglobulin G1 antibody that selectively inhibits gut lymphocyte trafficking [44]. Vedolizumab is typically administered intravenously, and a 3-dose regimen is associated with a lower relapse risk [45]. Because vedolizumab has gut tropism, it may have a reduced influence on the effect of cancer treatment and development [44].

Studies of infliximab have focused on its safety in non-cancer populations, and there is limited evidence guiding its use in cancer patients with colitis [38]. In contrast, vedolizumab, even as a long-term treatment, seems to have a favorable safety profile in cancer patients [46]. To alleviate infliximab’s adverse events while maximizing vedolizumab’s benefits, Zou et al. proposed a combination strategy employing rapid-induction infliximab plus maintenance vedolizumab [46]. We anticipate that the forthcoming prospective randomized clinical trial (NCT04407247) led by Wang et al. at MD Anderson, which compares infliximab and vedolizumab in cancer patients, will provide further insights into the drugs’ distinct contributions to cancer prognosis.

### Fecal microbiota transplantation

Studies suggest that the fecal microbiome influences the activity of ICIs and the development of ICI-induced colitis [47]. In patients with ICI-induced colitis refractory to intravenous steroids and biologics, fecal microbiota transplantation has elicited prompt clinical remissions. Wang et al. reported two cases of refractory ICI-induced colitis that were successfully treated with transplantations of fecal microbiota from healthy donors. Fecal microbiota transplantation should be considered for patients with ICI-induced colitis, especially those with challenging high-grade colitis, particularly if they have predictors of resistance, such as a lack of response to corticosteroid therapy within 72 h or endoscopic ulcerations [48]. Nevertheless, these findings require additional validation.

### Alternative treatments

Exploration of alternative treatments for ICI-induced colitis is underway. New potential options include calcineurin inhibitors like tacrolimus. Tacrolimus works by suppressing the release of the cytokines IL-2, TNF-α, and interferon γ, thereby inhibiting T cell activation and ultimately controlling colitis symptoms [49].

Mycophenolate mofetil, when added to steroids, has shown potential in reducing the duration of treatment by inhibiting T- and B-lymphocyte replication [50]. Other potential biologics for refractory ICI-related colitis include the IL-1 antagonist anakinra, and the anti–IL–12/IL-23 antibody ustekinumab. These drugs are designed to target the underlying drivers of the pathophysiological processes of irAEs [51].

Finally, promising molecules like Janus kinase inhibitors have also demonstrated efficacy in patients with ICI-induced colitis [48]. The exact impact of JAK-STAT inhibitors on ICI colitis remains unclear, but they may influence memory resident CD8 + T lymphocytes. Further research is needed, and ongoing JAK-STAT-based trials are expected to provide valuable insights. Randomized clinical trials have shown that JAK inhibitors can effectively control colonic inflammation and promote mucosal healing in moderate-to-severe IBD. JAK inhibitors like tofacitinib, upadacitinib, and filgotinib are approved for UC. While these results may not directly apply to ICI colitis due to different underlying causes, they suggest potential benefits of JAK-STAT pathway inhibition for ICI colitis. The ongoing TRICK study (NCT04768504), a phase 2 open-label trial, aims to assess tofacitinib’s efficacy in treating ICI colitis, potentially enhancing our understanding of this treatment approach [52].

## Current guidelines and best practice advice

The management of IMDC follows the guidelines from organizations such as the American Gastroenterological Association (AGA), the American Society of Clinical Oncology (ASCO), and the National Comprehensive Cancer Network (NCCN). A summary of best practice advice and recommendations is shown below:

- Infectious causes of diarrhea should be excluded first before treatment of suspected IMDC [53].
- Prompt diagnosis and treatment are required as symptoms and disease severity can escalate rapidly within days [53].
- Grade 1 diarrhea typically requires supportive treatment while continuing ICI therapy as the symptoms are generally mild and self-limited [54]:
  - Supportive treatment includes temporary use of antidiarrheal agents as needed, such as loperamide or cholestyramine. Patients are also recommended adequate oral hydration and dietary modifications with a lactose-free diet and reduced caffeine.
  - For symptoms persisting beyond 2 weeks, corticosteroid therapy may be considered; 9 mg of budesonide daily for at least 4 weeks is a common approach. Prednisone can be also administered for budesonide-resistant cases.
- For grade 2 or 3 diarrhea, ICI interruption is recommended, and hospitalization is necessary if systemic symptoms such as fever or dehydration are present. First-line treatment consists of systemic corticosteroid therapy that should be tapered over 4–6 weeks after symptom improvement. A return to grade 1 diarrhea is considered improvement [53]. Prednisone (1–2 mg/kg) is usually administered and has shown effectiveness in 87.5% of patients. Resuming anti-CTLA4 drugs after improvement is not recommended because of a high risk of recurrence, but anti-PD1/PDL1 drugs can be restarted as monotherapy [42, 54].
- For grade 4 diarrhea, ICIs should be stopped, hospitalization is required for monitoring, and intravenous corticosteroids should be started immediately. Intravenous methylprednisolone (1–2 mg/kg) is a standard practice [53].
- Patients with any diarrhea grade and corticosteroid resistance should be considered for escalation to biological agents (infliximab or vedolizumab) [53].
- Early stool testing for inflammatory markers such as lactoferrin and calprotectin may help stratify high-risk patients for endoscopic evaluation. Recommended in patients with grade 2 or higher diarrhea/colitis or selected patients with less severe symptoms [53].
- The diagnosis and severity of ICI-induced colitis should be confirmed by endoscopic evaluation before initiation of high-dose systemic glucocorticoids [53].
- In patients with predominant symptoms of pain, fever, or bleeding, abdominal imaging can be performed to exclude other serious complications. Abdominal imaging should not be performed routinely in patients presenting with diarrhea alone [53].
- Hospitalized patients should be reassessed daily to determine their eligibility for discharge. A patient can be discharged if they meet all of the following criteria: grade 1 diarrheal symptoms (< 4 stools per day), resolution of severe abdominal pain, tolerance of oral diet, and normalization of vital signs [55].

**Table 2:** Management of immune-mediated diarrhea and colitis

Grade*	Symptoms	Management	ICI status
1	**Diarrhea:** increase of < 4 stools/day **Colitis:** asymptomatic	Supportive treatment (antidiarrheal, hydration, diet modifications) CS if symptoms > 2 weeks	May continue without interruption
2	**Diarrhea:** increase of 4–6 stools/day **Colitis:** watery diarrhea, cramping, abdominal pain, blood and mucus in stool	Systemic CS (prednisone 1–2 mg/kg) Hospitalization if systemic symptoms (e.g., fever, dehydration)	Interruption recommended May resume anti-PD1/PDL1 only after improvement to grade 1 or less
3	**Diarrhea**: increase of ≥ 7 stools/day **Colitis:** same as grade 2 with fever, ileus, or peritoneal signs		
4	**Diarrhea:** Life-threatening consequences **Colitis:** same as grade 3 with ischemic bowel, perforation, or toxic mega-colon	IV CS (methylprednisolone 1–2 mg/kg) Hospitalization	Permanent interruption
5	**Diarrhea and colitis:** death	-	-

*Based on Common Terminology Criteria for Adverse Events (CTCAE) as historically used. *CS*, corticosteroids; *ICI*, immune checkpoint inhibitor; *IV*, intravenous; *PD1/PDL1*, programmed cell death 1/programmed cell death ligand 1

The management structure, according to AGA, ASCO, and NCCN guidelines, and CTCAE diarrhea/colitis grade, is summarized in Table 2**.**

## Other gastrointestinal irAEs associated with ICIs

In a meta-analysis by Liu et al., the most prevalent gastrointestinal adverse event of any grade was diarrhea, followed by nausea, decreased appetite, vomiting, constipation, colitis, and abdominal pain [4]. Nausea and vomiting are typically mild, especially when they occur in isolation, but their severity may escalate when they occur alongside conditions like infections, endocrinopathies, or organ damage. However, grade 3 or higher nausea and vomiting are seen in only 2% of cases [56].

Documented cases of visceral hypereosinophilia linked to ICIs are uncommon. Eosinophil counts may become high enough that complications related to hypereosinophilic visceral involvement can arise [57]. One manifestation of visceral hypereosinophilia as an irAE is gastrointestinal eosinophilia, a rare condition characterized by eosinophilic infiltration in the gastrointestinal tract wall and an absence of other established causes of tissue eosinophilia [58]. Evidence suggests that eosinophilia can persist for up to several months, even after the discontinuation of ICIs [59]. In one study, 30% of patients who received ipilimumab for malignant melanoma developed hypereosinophilia. Among these patients, 77% experienced ICI-associated irAEs, which were predominantly gastrointestinal irAEs in the form of diarrhea and colitis [60].

Rare cases of celiac disease emergence have been reported, but the connection between celiac disease and ICI treatment lacks clear evidence In the majority of reported cases, adopting a gluten-free diet proved effective in reversing the damage, and therapeutic intervention was not required [61].

Remarkably, small bowel involvement due to ICIs has been recognized as villous atrophy or as generally severe ulcerative enteritis without any indication of villous atrophy. The ulcerative phenotype is occasionally accompanied by significant gastrointestinal bleeding or perforation of the small intestine [62]. The villous atrophy phenotype may resemble celiac disease, with elevated intraepithelial lymphocyte counts in the duodenum, but it is distinguished by the presence of neutrophilic infiltrates or erosions. For this reason, a gluten-free diet may be helpful, and symptoms usually resolve after therapy. Mukewar et al. suggested using open-capsule budesonide and infliximab to treat these adverse events [63].

Hepatitis as a side effect occurs in 19% of patients receiving both anti-CTLA4 and anti-PD1 ICIs [64]. Patients’ symptoms range from modest transaminase elevation to fulminant liver failure. The predominant damage is hepatic, but cholangitis and cholecystitis have also been documented [11].

Following ICI administration, 2–8% of patients have elevated amylase and lipase levels. Elevated lipase levels have been used to define type 3 autoimmune pancreatitis; typical pancreatitis with radiological criteria is less frequent [56]. However, no studies have definitively established ICIs as a cause of elevated amylase or lipase levels [65].

## Conclusion

Gastrointestinal irAEs remain a common complication of ICIs with diarrhea and colitis among the most prevalent side effects. Their incidence is higher in patients receiving anti-CTLA4 compared to anti-PD1/PDL1. Risk factors for developing IMDC include baseline gut microbiota, preexisting autoimmune disorders such as IBD, and type of neoplasm. The hallmark symptom of colitis is diarrhea which may be accompanied by mucus or blood in stools. Patients may also experience abdominal pain, fever, vomiting, and nausea. A comprehensive differential diagnosis involves a set of laboratory tests, analyzing drug history, temporal associations, specific histological features, and multiple organ involvement to ensure accurate identification. Treatment should be initiated promptly to avoid serious complications. Current management is based on corticosteroids as first-line, and immunosuppressants like infliximab or vedolizumab for refractory cases. Microbiota transplantation and specific cytokines and lymphocyte replication inhibitors are being investigated. However, existing data are primarily from case reports, and further studies to assess drug usefulness in IMDC are warranted.

## Data Availability

No datasets were generated or analysed during the current study.
